# Placenta Praevia

**Published:** 1884-11

**Authors:** 


### Placenta Praevia.

Dr. E. P. Christian, of Wyandotte, Wis., reports (*Medical Record*) eight cases of placenta praevia, with statistics from several sources. He considers placenta praevia a frequent cause of abortions in the early months of pregnancy. *Vice versa*, he considers frequent abortions a fruitful cause of placenta praevia, or of the subinvoluted state of the uterus, which predisposes to placenta praevia.

